# Normal Alpha-1-Antitrypsin Variants Display in Serum Allele-Specific Protein Levels

**DOI:** 10.1021/acs.jproteome.2c00833

**Published:** 2023-03-22

**Authors:** Shelley Jager, Dario A. T. Cramer, Albert J. R. Heck

**Affiliations:** † Biomolecular Mass Spectrometry and Proteomics, Bijvoet Center for Biomolecular Research and Utrecht Institute for Pharmaceutical Sciences, University of Utrecht, Padualaan 8, Utrecht 3584 CH, The Netherlands; ‡ Netherlands Proteomics Center, Padualaan 8, Utrecht 3584 CH, The Netherlands

**Keywords:** alpha-1-antitrypsin, genotypes, proteogenomics, allele-specific protein serum levels, gene variants, native mass spectrometry, biomarkers, alpha-1-antitrypsin deficiency

## Abstract

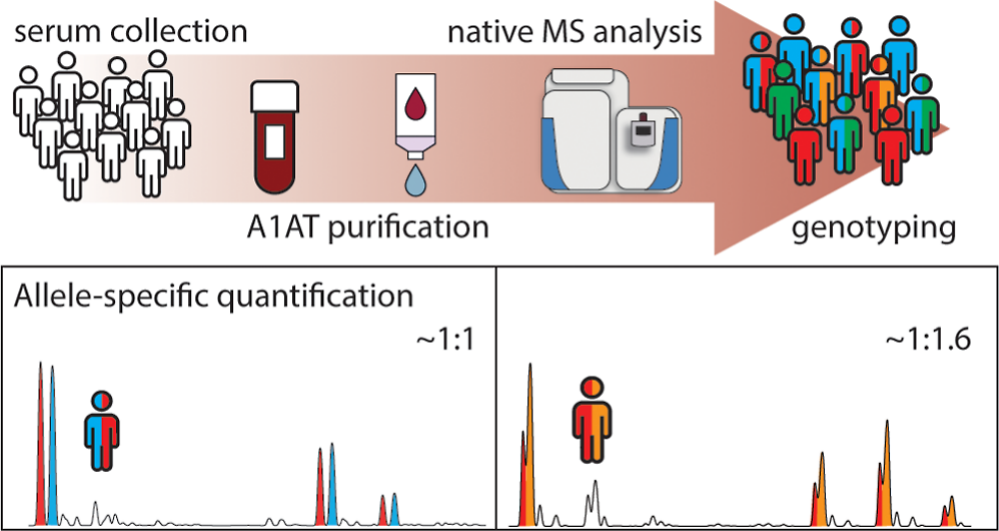

Alpha-1-antitrypsin (A1AT or SERPINA1) has been proposed as a putative biomarker distinguishing healthy from diseased donors throughout several proteomics studies. However, the SERPINA1 gene displays high variability of frequent occurring genotypes among the general population. These different genotypes may affect A1AT expression and serum protein concentrations, and this is often not known, ignored, and/or not reported in serum proteomics studies. Here, we address allele-specific protein serum levels of A1AT in donors carrying the normal M variants of A1AT by measuring the proteoform profiles of purified A1AT from 81 serum samples, originating from 52 donors. When focusing on heterozygous donors, our data clearly reveal a statistically relevant difference in allele-specific protein serum levels of A1AT. In donors with genotype PI*M1VM1A, the experimentally observed ratio was approximately 1:1 (M1V/M1A, 1.00:0.96 ± 0.07, *n* = 17). For individuals with genotype PI*M1VM2, this ratio was 1:1.28 (M1V/M2, 1.00:1.31, ±0.19, *n* = 7). For genotypes PI*M1VM3 and PI*M1AM3, a significant higher amount of M3 was observed compared to the M1-subtypes (M1V/M3, 1.00:1.84 ± 0.35, *n* = 8; M1A/M3, 1.00:1.61 ± 0.33, *n* = 5). We argue that these observations are important and should be considered when analyzing serum A1AT levels before proposing A1AT as a putative serum biomarker.

## Introduction

Alpha-1-antitrypsin (A1AT), also known as SERPINA1, is one of the important circulating anti-proteases abundantly present in human blood (∼1 g/L) and other body fluids.^1,2^ A1AT is mainly synthesized in the liver, after which it is secreted into the circulation. It is involved in a variety of anti-proteolytic processes, albeit that its major function is to inhibit neutrophil elastase in the lung.^3^ In addition, A1AT is an acute phase protein involved in a variety of anti-inflammatory and immunomodulatory events.^4−8^ A1AT serum levels increase within hours after inflammation or infection and affect the signaling of several types of immune cells, including B-cells, T-cells, red blood cells, and neutrophils.^9^ Furthermore, the proteoform profile of A1AT, mainly the *N*-glycosylation patterns, may change substantially upon inflammation.^10−15^

**Figure 1:**
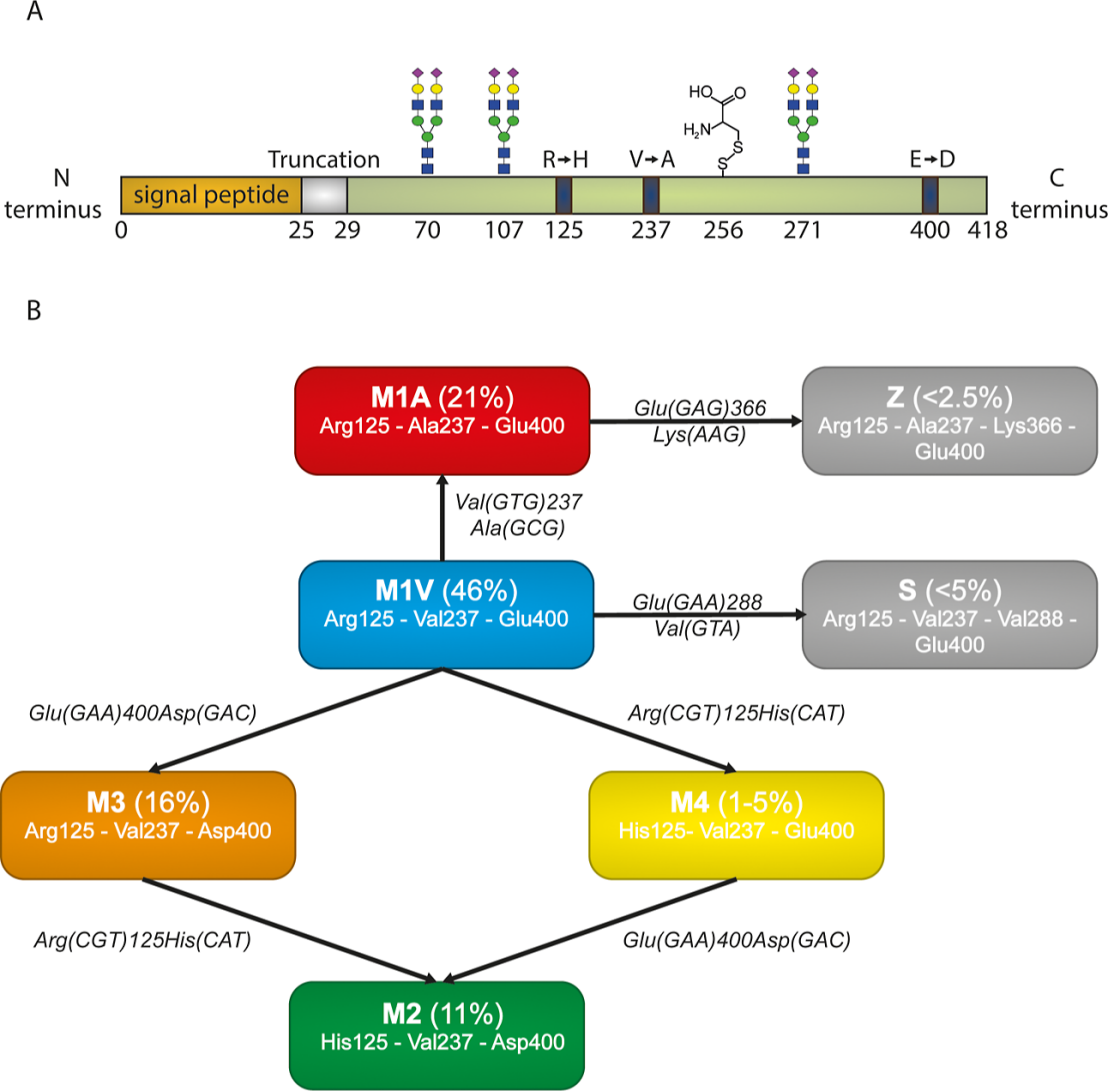
Sequence, post-translational modifications, and most prevalent “normal” haplotypes of A1AT. (A) An overview of the A1AT sequence, with in orange the signal peptide and in green the mature circulating protein. Indicated are the glycosylation sites, the cysteinylation site, and the mutation hotspots (dark blue) for the normal M1A, M1V, M2, M3, and M4 haplotypes. Indicated in gray are the five amino acids that are occasionally missing in serum A1AT, forming a truncated form of A1AT. (B) Flowchart of some of the most prevalent normal haplotypes of A1AT. In bold is the name of the haplotype, and in between brackets is the approximated prevalence in the Caucasian population.^21,22^ The change of the amino acid and the codon is indicated in italics next to the arrow, as well as the codon sequence in between brackets.

The gene for A1AT encodes a 418 amino acid protein, of which the first 24 amino acids represent the signal peptide that is cleaved off in the maturation of the protein (Figure 1A). The structural features of “normal” A1AT have been well studied and several post translational modifications (PTMs) have been annotated and identified. There are three *N*-glycosylation sites (N70, N107, and N271), which are usually occupied with complex type *N*-glycans.^16^ Furthermore, A1AT has a free cysteine that is often cysteinylated (C256) and it can be (to a variable degree) truncated at its *N*-terminus (deletion of the *N*-terminal EDPQG stretch)^17^ (Figure 1A).

In humans, over 150 genetic variants of this protease inhibitor (PI) have been reported.^18^ Allelic variants have traditionally been classified based on their migration behavior on isoelectric focusing (IEF) gels, with the M alleles migrating in the middle.^1^ All faster migrating variants have been named A–L; all slower migrating variants have been named N–Z. Several of the more prevalent PI*M subtypes are considered to represent the healthy, or normal, A1AT variants. Haplotypes PI*S (Glu288Val) and PI*Z (Gly366Lys), not that frequently present in the population, are associated with A1AT deficiency that may result in, among others, lung disease (e.g., COPD) or liver disease.^3,19,20^

A variety of subtypes exist among these “normal” M alleles, with the most common (prevalence of approximately 46% in Caucasians) allelic form being referred to as PI*M1V.^23,24^ Other prevalent haplotypes are as follows: PI*M1A (Val237Ala) with a prevalence of approximately 21%, PI*M2 (Arg125His and Glu400Asp) with a prevalence of 16%, and PI*M3 (Glu400Asp) with a prevalence of 11%.^21,23−25^ To illustrate this further, an overview of the most common haplotypes and their associated mutations is depicted in Figure 1B. The A1AT alleles are co-dominantly expressed, and with four M variants with a prevalence higher than 10%, this leads to high genetic variety across individuals. A heterozygous individual with both an M1V and M1A allele would then be described as the PI*M1VM1A genotype.

Serum concentrations of A1AT (or SERPINA1) are assessed clinically, primarily to discover individuals with severe A1AT deficiency.^26^ It is known that serum concentrations of A1AT can be influenced by genetics. A small percentage of individuals, ranging from 1/1500 to 1/10,000 depending on ethnicity, exhibit severe A1AT deficiency due to homozygosity or compound heterozygosity for deficiency (most commonly, Z and S alleles, and other rare allelic variants) or Null alleles.^26,27^ However, it has been generally assumed (so far) that the common subtypes of the M alleles (M1-4) have little influence on A1AT serum concentrations.

We previously demonstrated that native mass spectrometry (MS) can be used to qualitatively and quantitatively measure the proteoform profiles of A1AT purified from serum.^28,29^ Here, we applied this method to a variety of individual serum samples (*n* = 81), both from healthy donors as well as different patients, obtained from variable origins. The high-resolution native mass spectra allow us, due to the small mass differences caused by the underlying mutations, to distinguish and assess the relative genotype specific serum concentrations of A1AT in heterozygous individuals. Markedly, we observe that these concentrations are not identical, whereby especially the Glu400Asp mutation and to a lesser extent the Arg125His mutation seem to lead to an increased serum abundance relative to either M1-haplotype. We argue that this is important information as it could affect both serum proteomics as well clinical diagnostic measurements.

## Materials and Methods

### Donor Population

**Figure 2:**
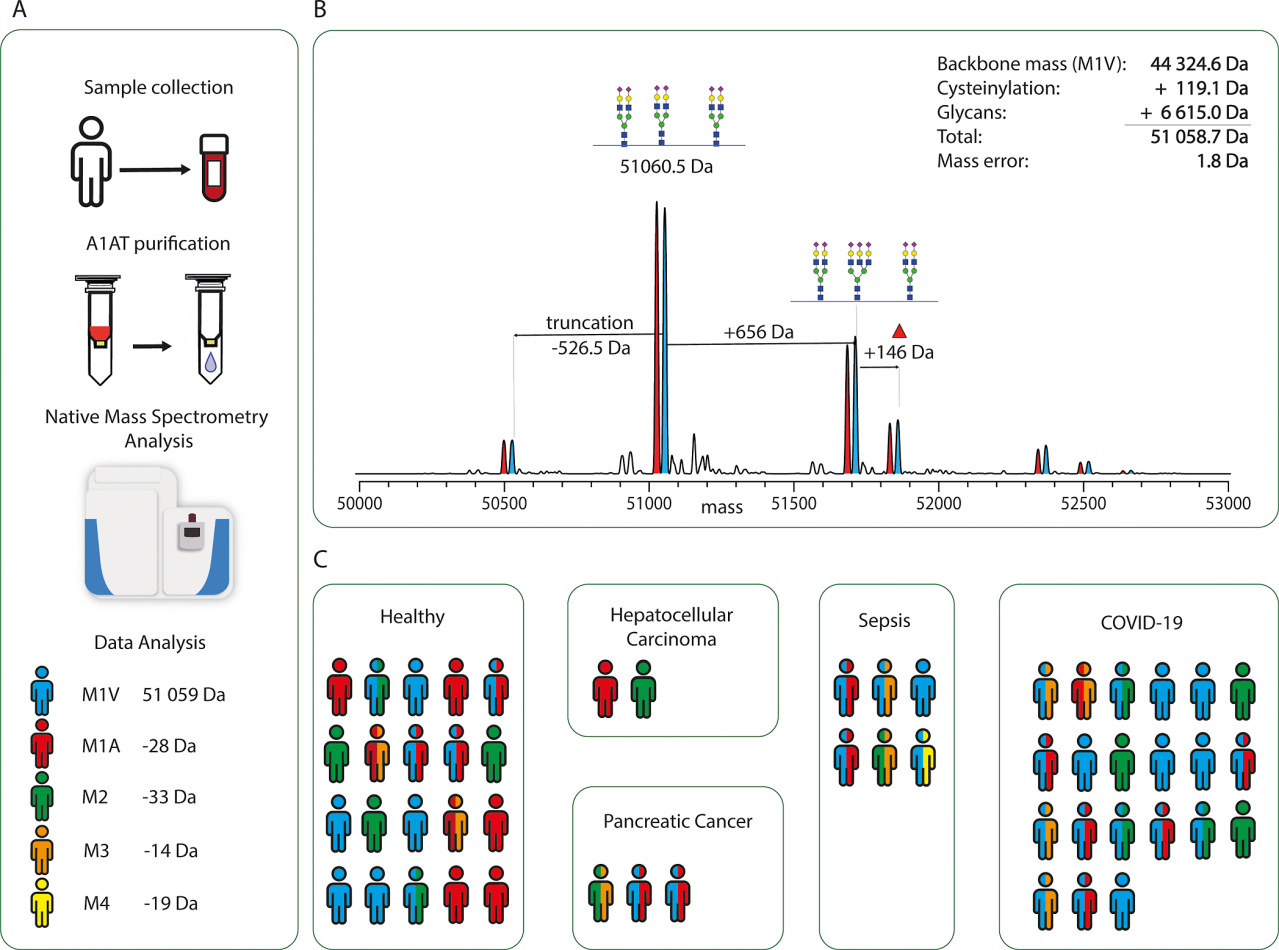
A) Experimental setup for A1AT purification and analysis of individual donor serum samples. (B) Annotated proteoform profile of serum A1AT resulting from the zero-charge deconvoluted native mass spectrum. Peaks are annotated with the most probable glycan composition as described earlier.^10,17^ Additionally, theoretical mass shifts corresponding to these PTMs are given. (C) Overview of all donors, and their genotypes, as present in this study, grouped by their reported medical condition. Figurine color corresponds to the donor’s genotype using the color scheme depicted in A. Double colored figurines represent heterozygous donors.

We collected in total 81 serum samples, originating from 52 individual donors (Figure 2C). We used a random selection of serum samples, also used in previous studies from our laboratory, originating from various cohorts, to increase our chance of including donors being heterozygote for A1AT. Most of the healthy serum samples were provided by the Sanquin Institute (The Netherlands). Two other healthy serum samples were obtained from Discovery Life Sciences (USA) as well as several serum samples of pancreatic adenocarcinoma, hepatocellular carcinoma, and sepsis patients. Additionally, samples from a cohort of patients with SARS-CoV-2 infection were included, detailed information about this cohort has been previously described.^30^ All serum samples were stored at −80 °C until use.

### Purification of Alpha-1-Antitrypsin from Serum

Unless stated otherwise, all other chemicals and proteins were purchased from Sigma-Aldrich (Saint Louis, MO, USA). Serum samples were filtered using a 0.22 μm filter (WAT200516 Acrodisc, Waters, Milford, MA, USA), aliquoted, and then stored at −80 °C until use. Alpha-1-antitrypsin was purified from 25 to 100 μL of serum of each individual donor as described earlier.^28^ In short, empty spin columns were conditioned with PBS, and packed with antitrypsin affinity resin (CaptureSelect Alpha-1 Antitrypsin Affinity Matrix, Thermo-Fisher, Waltham, MA, USA), and incubated with serum for 1 h at RT. Next, the column was washed three times with PBS and A1AT was eluted using glycine (0.1 M, pH 3.0) which was immediately quenched with TRIS/HCl (1 M, pH 8.5).

### Native MS Analysis of Purified A1AT

20–30 μL of purified A1AT was buffer exchanged into 1.5 M aqueous ammonium acetate (AMAC) (pH 7.2) by ultrafiltration with a 10 kDa cutoff filter (vivaspin500, Sartorius Stedim Biotech, Germany). The final volume was 15–30 μL. Samples were diluted or concentrated accordingly to achieve the strongest signal. The samples were analyzed on a modified Exactive Plus Orbitrap instrument with extended mass range (EMR) (Thermo Fisher Scientific, Germany), a standard *m/z* range of 500–15.000 was used, as has been previously described.^31^ The voltage offsets on the transport multipoles and ion lenses were manually tuned for optimal transmission of protein ions. Nitrogen was used in the higher-energy collision dissociation (HCD) cell at a gas pressure of 6–8 × 10^–10^ bar. The spray voltage was set to 1.32 kV; the in-source collision-induced fragmentation energy and the collision energy were set to 20 and 30 eV, respectively, and the resolution was 35,000 (@ *m/z* 200). Prior to use, the instrument was calibrated using a CsI solution.

### Native MS Data Analysis

The electrospray ionization (ESI) mass spectrum was deconvoluted to a zero-charge spectrum using Intact Mass software from Protein Metrics (version 4.0–43 × 64) to extract the accurate masses of the A1AT proteoforms. Used deconvolution settings were as follows: mass range 50,000–55,000 Da; *m*/*z* range 200–4500 min difference between peaks 7 Da, iteration 20–30, and charge range 5–20. PTMs were analyzed manually and glycan structures were assigned based on previously proposed structures.^16^ Average masses used to assign these PTMs were as follows: hexose/mannose/galactose (Hex/Man/Gal, 162.1424 Da), *N*-acetylhexosamine/*N*-acetylglycosamine (HexNac/GlcNAc, 203.1950 Da), deoxyhexose/fucose (dHex/Fuc, 146.1430 Da), cysteinylation (Cys, 119.1421 Da), and the *N*-terminal truncation (−EDPQG, −526.50 Da).

To calculate the relative abundances per haplotype, the Intact Mass output was taken and a selection of frequent occurring proteoforms were assigned automatically using an in-house written script in R (version 4.2.0) (Figure S1). Overlapping proteoforms were excluded from quantification. Assigned peaks were validated manually. Relative haplotype abundances were calculated by normalizing for the PI*M1V abundance for the following genotypes: PI*M1VM1A, PI*M1VM2, and PI*M1VM3. For the genotype PI*M1AM3, the abundances were normalized for the PI*M1A abundance. The statistical significance was tested using an unpaired *T*-test. Calculations and bar graphs were made using *R*.

## Results

### High-Resolution Native MS Analysis Reveals Comprehensive A1AT Proteoform Profiles

To obtain a sufficient amount of samples to cover most common genotypes, a random cohort of samples was constituted, consisting of serum samples from 52 donors. For a few donors, samples were collected over multiple time-points, leading to a total of 81 serum samples analyzed. For each of these samples, A1AT was purified using affinity chromatography, as described earlier.^28^ Purified A1AT was analyzed by high-resolution native mass spectrometry, after which the mass spectrum was assigned based on theoretical knowledge of the sequence of A1AT and the possible PTMs, and the donor was genotyped accordingly (Figure 2A).

The recorded native MS spectra usually showed ion signals originating from A1AT in three charge states ranging from [M + 13H]^13+^ to [M + 15H]^15+^ with each charge state containing various ion series originating from the different haplotypes and (glyco)proteoforms (Figure 2B). Usually, the most abundant peak had a mass corresponding to the A1AT amino acid backbone mass +119 Da (cysteinylated cysteine) + 6615 Da (corresponding to 3*x* bi-antennary complex glycan). In Figure 2B, a typical deconvoluted native MS spectrum of A1AT is shown, extracted from a donor heterozygous for A1AT (PI*M1VM1A). Typical for these heterozygous A1AT profiles is the double peak pattern, for each individual resolved haplotype in each different proteoform. This spectrum displays large similarities with earlier annotated proteoform profiles of A1AT as reported by others^16,32−34^ and us,^28,29^ and therefore, we do not discuss these profiles here further in detail. All samples were genotyped based on the theoretical mass difference between M1V and the other M variants, which revealed a total of 27 heterozygous donors and 25 homozygote donors. For some donors, we had samples over two or three time-points, which led to a total of 39 samples from heterozygote donors (Figure 2C).

### A1AT Haplotype Affected Abundances in Heterozygote Donors

**Figure 3:**
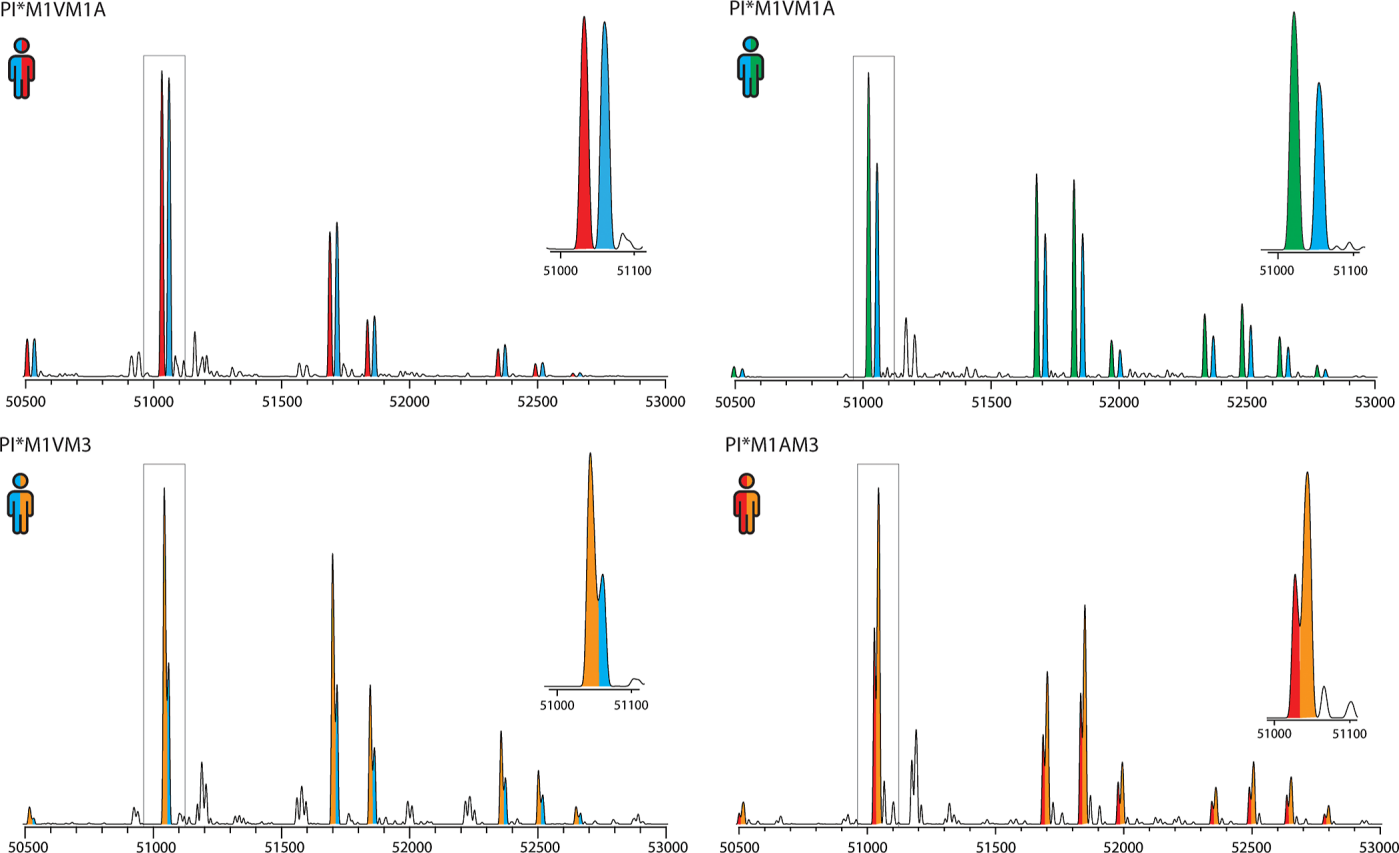
Illustrative examples of deconvoluted native mass spectra of serum A1AT obtained from heterozygote donors. Examples from the four most frequent heterozygote combinations found in our cohort are depicted. Peaks corresponding to each genotype are colored as follows: PI*M1V is blue, PI*M1A is red, PI*M2 is green, and PI*M3 is orange. The most abundant peak combination is enlarged in the right corner. For the donor with PI*M1VM1A (left top), it is clearly evident that each split paired peak is of equal abundance, while for donors of other heterozygote combinations, this is not the case.

Next, we focused on all samples originating from donors being heterozygote for A1AT since we noticed that the protein levels corresponding to different A1AT haplotypes seemed not always equally distributed in those samples (Figures 3 and S1). Samples from individuals whose serum had been collected over multiple time points were regarded as separate samples. Healthy and patient (sepsis, hepatocellular carcinoma, pancreatic adenocarcinoma, or COVID-19) samples were combined in this analysis as we hypothesized that the haplotype affected abundances were not affected by the medical condition of the donor. A distribution of all the distinct haplotypes identified by using native MS is summarized in Table S1. Notably, heterozygous donors for PI*M1AM2 were not found in our analysis. The average mass difference between these two haplotypes is just 5,02 Da. Unfortunately, these haplotypes can therefore not be resolved as separate peaks with the mass resolution obtained; individuals with haplotype PI*M1AM2 will thus be indistinguishable from M1A or M2 homozygote donors.

**Figure 4:**
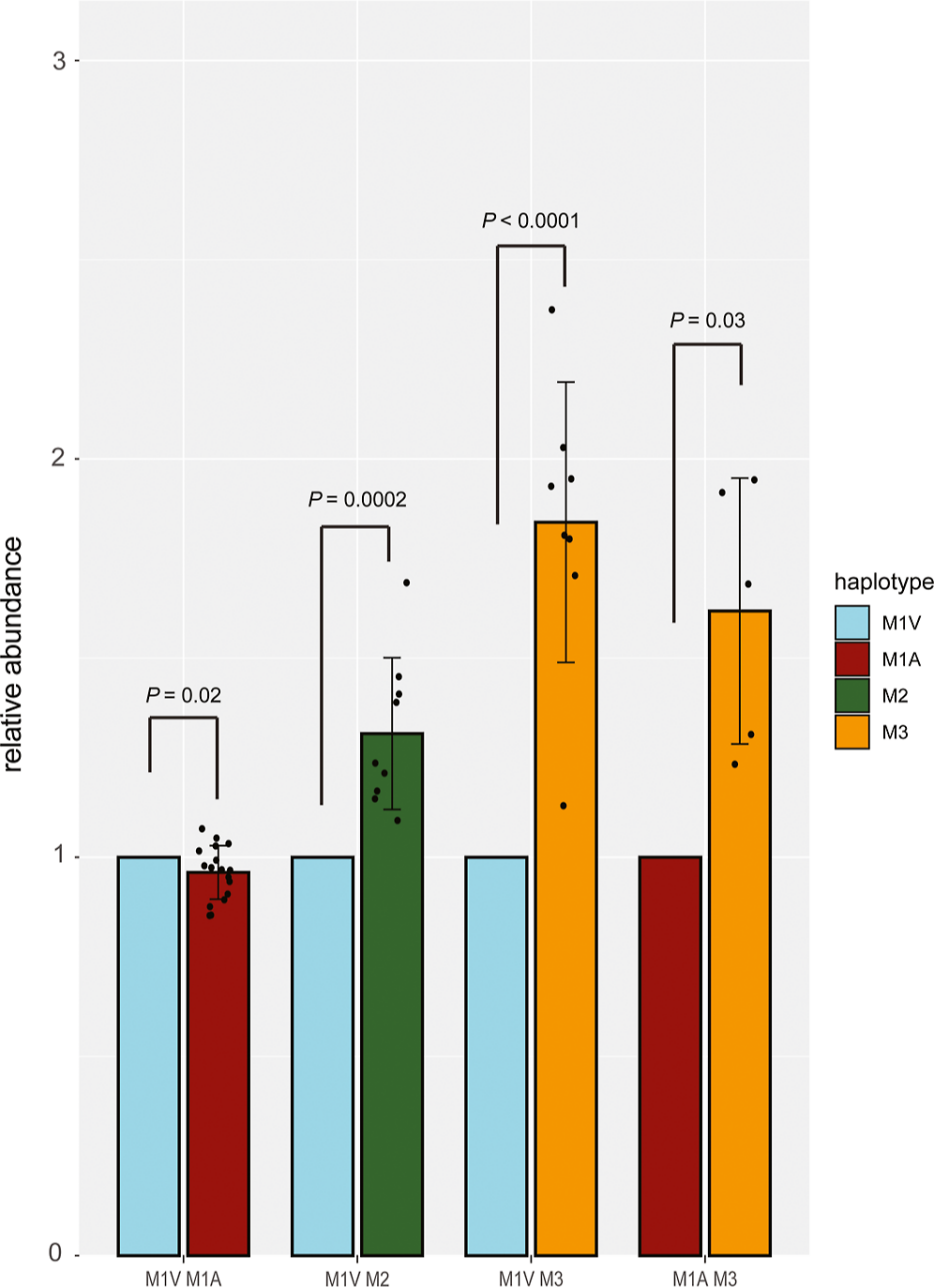
A1AT abundance in serum is affected by haplotypes in heterozygous individuals. Relative abundance was calculated by taking the sum of the intensities of all annotated peaks corresponding to the haplotypes (as described in the Methods) and subsequent normalization to either the total abundance of M1V (for genotypes PI*M1VM1A, PI*M1VM2, and PI*M1VM3), or normalized to the total abundance of M1A (for genotype PI*M1AM3). In the heterozygous samples with haplotype PI*M1VM1A, the observed ratio was approximately 1:1 (M1V/M1A, 1.00:0.96 ± 0.07, *n* = 17). For individuals with haplotype PI*M1VM2, this ratio was 1:1.31 (M1V/M2, 1.00:1.31, ±0.19, *n* = 9). For haplotypes PI*M1VM3 and PI*M1AM3, a significant higher amount of M3 was observed compared to the M1-subtypes (M1V/M3, 1.00:1.84 ± 0.35, *n* = 8; M1A/M3, 1.00:1.61 ± 0.33, *n* = 5).

First, we extracted that the haplotype ratio in A1AT serum abundances in heterozygous samples with haplotype PI*M1VM1A is approximately 1:1 (M1V/M1A, 1.00:0.96 ± 0.07, *n* = 17) (Figure 4). In individuals with genotype PI*M1VM2, a relative higher serum abundance, statistically relevant, of haplotype PI*M2 was observed (M1V/M2, 1.00:1.31, ±0.19, *n* = 9). For both genotypes PI*M1VM3 and PI*M1AM3, a significant higher amount of haplotype M3 was observed compared to the M1-subtypes (M1V/M3, 1.00:1.84 ± 0.35, *n* = 8; M1A/M3, 1.00:1.61 ± 0.33, *n* = 5). The PI*M2M3 genotype was only observed in two donors, whereby the A1AT concentrations for both haplotypes were equal in the serum samples we analyzed (Table S1, sample numbers 23 and 30; Figure S1H and N). The PI*M1VM4 haplotype was identified in just one donor, the distribution of haplotypes was also equally distributed. Unpaired *T*-tests were performed to statistically compare the serum concentration of the two haplotypes within a single genotype. Although the cohort is rather small, the extracted *p*-values were significant: *p* = 0.0002, *p* = 0.003, and *p* < 0.0001 for PI*M1VM2, PI*M1AM3, and PI*M1VM3, respectively, in contrast to a *p*-value of 0.03 for PI*M1VM1A. Samples from which multiple time-points were available, revealed that the relative haplotype linked concentrations of A1AT in serum remained very consistent over time.

## Discussion

Here, we measured allele-specific protein serum levels directly at the intact protein level, focusing on a key polymorphic serum protein, namely, alpha-1-antitrypsin (A1AT). A priori, we expect that there may be several factors involved in allele-specific protein expression/abundances, including potential differences in serum half-lives and differences in secretion levels. Evidently, the genetic aspect is a key factor. Understanding the genetic basis of gene regulatory variation has been a prime goal of evolutionary (and medical) genetics.^35^ Regulatory variation can act in an allele-specific manner (*cis*-acting) or it can affect both alleles of a gene (*trans*-acting). While microarrays and high-throughput sequencing have enabled genome-wide measurements of allele-specific expression (ASE) on the transcript level, methods for measurement of protein ASE (pASE) lag behind both in throughput and in accuracy.^36^ A few other studies have been reported, measuring pASEs using standard peptide-centric LC–MS approaches.^37,38^ This would require additional optimization as one needs to identify the peptides harboring the allele specific mutations. Therefore, these mutations then need to be in “ideal” tryptic peptides that fly and fragment well inside the mass spectrometer. This can be somewhat circumvented as demonstrated by Shi et al. who developed an elegant targeted proteomics method for the quantification of allele-specific protein expression based on scheduled parallel reaction monitoring (PRM) using a heavy stable isotope-labeled concatemer.^39^ Although powerful, peptide-centric analysis may be hindered by changes in allele-specific peptides, which for A1AT occurs, for instance, for peptides covering the Arg125His mutations, as Arg125 would be an expected tryptic cleavage site, which is no longer present after the Arg125His mutation. The use of different proteases might be needed to fully cover all frequently occurring mutations. Additionally, due to the high variability of A1AT genotypes within the general population and the possibility of co-modifications (for example in PI*M3), protein quantification at the peptide level would be a very difficult task.

Here, we used a more direct, and less biased approach by measuring comprehensive proteoform profiles of intact A1AT proteins, purified from as little as 25 μL of serum. Although we acknowledge that this method is not suitable for all possible heterozygous combinations of M-haplotypes, as higher mass resolution is required to resolve individual haplotypes with smaller mass differences (for example for PI*M1AM2, with a Δ*m* of 5 Da). However, this method enabled for the extraction of quantifiable information on the haplotypes of six different heterozygous combinations.

The observations made here that the ratio between serum protein levels of A1AT originating from distinct haplotypes in heterozygous individuals is unequal is not unprecedented, as this is well known for patients suffering from anti-trypsin deficiency.^3^ The A1AT deficiency alleles cause severely decreased levels of circulating A1AT.^1,40^ The PI*S (Glu288Val) variant generates 60% of the circulating A1AT levels, as compared to the M variants; while the PI*Z (Glu366Lys) variant generates only 10–15%.^1,26,41^ Thus, heterozygotes for the PI*MS haplotypes will have on average diminished circulating A1AT levels in serum of ∼80% (50% from the PI*M and 30% from the PI*S); heterozygotes carrying PI*MZ of ∼55% (50% from PI*M, 5–7.5% from PI*Z). For the Z allele, the lesser amount of A1AT in the circulation is largely caused by polymerization and subsequent accumulation in the endoplasmic reticulum.^42^ While for the S allele, this is thought to be caused by intracellular degradation prior to protein excretion.^43^

Although these lowered abundances in patients carrying PI*S and PI*Z alleles have been well described and documented, it has been generally assumed that the subtypes of the more frequently present “normal” M alleles have no influence on A1AT serum concentrations. In contrast, our results reveal that the serum levels of A1AT variants from normal M mutations (Arg125His and Glu400Asp) are increased relatively to PI*M1V and PI*M1A. In the case of PI*M3 its abundance can even be twice as high as their M1-counterpart. However, we still see quite a spread between samples, especially for the PI*M3 heterozygotes, and it is not clear whether this spread is influenced by the medical variety between donors or whether it is the result of a low sample size.

With their relative abundance differences, it would also be expected that these variants (PI*M2 and PI*M3) cause an overall increase of circulating A1AT. However, it is difficult to prove this directly in homozygote donors, partly due to the limited number of healthy donor samples we had available, but even more so because of high inter-donor variability in serum A1AT. Having sera from both healthy and diseased donors, we suspect that the unequal A1AT concentrations of haplotypes is genetically defined and not substantially affected by the healthy/disease state.

Because haplotype PI*M2 has both the Arg125His and the Glu400Asp mutation, and PI*M3 has only the Glu400Asp mutation; it is especially interesting to also look at heterozygous individuals with the PI*M4 haplotype (Arg125His). In this sample set, the PI*M4 haplotype was identified in only one heterozygous individual; the abundance seemed rather equal to that of PI*M1V. This suggests that the increased abundances measured here might be primarily linked to the Glu400Asp mutation, but due to the low number of individuals with the PI*M4 haplotype (*n* = 1), this must be confirmed.

As this is the first time such differences in serum concentrations from normal M mutations are reported, neither the cause nor the effect of this is yet known. A recently published study examined the binding of A1AT to elastase with regard to its glycan composition, comparing PI*M1V to PI*M3 and found that core fucosylation might stabilize elastase binding in PI*M1V, but not in PI*M3.^34^ However, this study focused more on glycosylation differences than the binding differences caused by genetic polymorphism. Further studies may elucidate on the combined effect of different binding affinities between genetic variants and glycostructures, and the increased or decreased abundance of these specific polymorphic variants.

Additionally, our findings are particularly relevant for serum proteomics studies that have identified A1AT as a potential biomarker for the disease state, by observing changes in abundance between health and disease state in the range we observe here to be potentially the same as haplotype induced changes (i.e., 1.5–2.5 fold increase). Many of these studies just measure A1AT abundance, without knowing or defining the donors A1AT haplotypes. We found numerous examples of such studies. For example, Li et al., quantified by iTRAQ- 2DLC-MS/MS the A1AT levels in donors with or without sepsis reporting a 2.54 increase in sepsis patients.^44^ Additionally, in several recent serum proteomics studies on severe COVID patients, including from our own laboratory, the A1AT (SERPINA1) levels have been reported to be different between survivors and non-survivors, often also in a range of 1.5–2.5 fold, but again, these data are reported without reporting or knowing the donors A1AT haplotypes.^30,45,46^ The here reported data make clear that future serum proteomics studies need to take into account the proteogenomic aspects of the reported proteins, as not only A1AT but also many other serum proteins have highly frequent haplotypes, including, for instance, fetuin,^47^ histidine-rich glycoprotein and haptoglobin.^48^

The data presented here reveal how individual profiling at the intact protein level by native mass spectrometry may elucidate allele-specific protein serum levels directly at the intact protein level and additionally provide how this may also influence the proteins’ post-translational modification, as previously shown for fetuin.^47^ Although at present this is not yet a high-throughput method, it is evident that such information is essential to make proposed serum protein biomarkers more endorsed.

## Supporting Information Available

Annotated proteoform profiles of donors heterozygous for alpha-1-antitrypsin and haplotype distribution and medical condition of each donor (PDF)

## Notes

The data sets presented has been deposited to the MassIVE repository, Accession number MSV000090934: ftp://massive.ucsd.edu/MSV000090934/.
