# Supplementary material for: Normal Alpha-1-Antitrypsin Variants Display in Serum Allele-Specific Protein Levels
Source: J Proteome Res. 2023 Mar 22;22(4):1331–8. doi: 10.1021/acs.jproteome.2c00833 (PMC10088046; doi:10.1021/acs.jproteome.2c00833)
Supplement: Supplementary file 1 — pr2c00833_si_001.pdf [file pr2c00833_si_001.pdf]

Normal Alpha-1-Antitrypsin variants display in serum allele-specific protein levels

Shelley Jager[1,2], Dario A.T. Cramer[1,2] and Albert J.R. Heck*[1,2]

[1] Biomolecular Mass Spectrometry and Proteomics, Bijvoet Center for Biomolecular Research and Utrecht Institute for Pharmaceutical Sciences, University of Utrecht, Padualaan 8, Utrecht 3584 CH, The Netherlands

[2] Netherlands Proteomics Center, Padualaan 8, Utrecht 3584 CH, The Netherlands

Table of contents

	Page number
**Supplemental Figure S1: Annotated proteoform profiles of donors heterozygous for alpha-1-antitrypsin.**	S-2
**Supplemental Table 1: Haplotype distribution and medical condition of each donor**	S-19

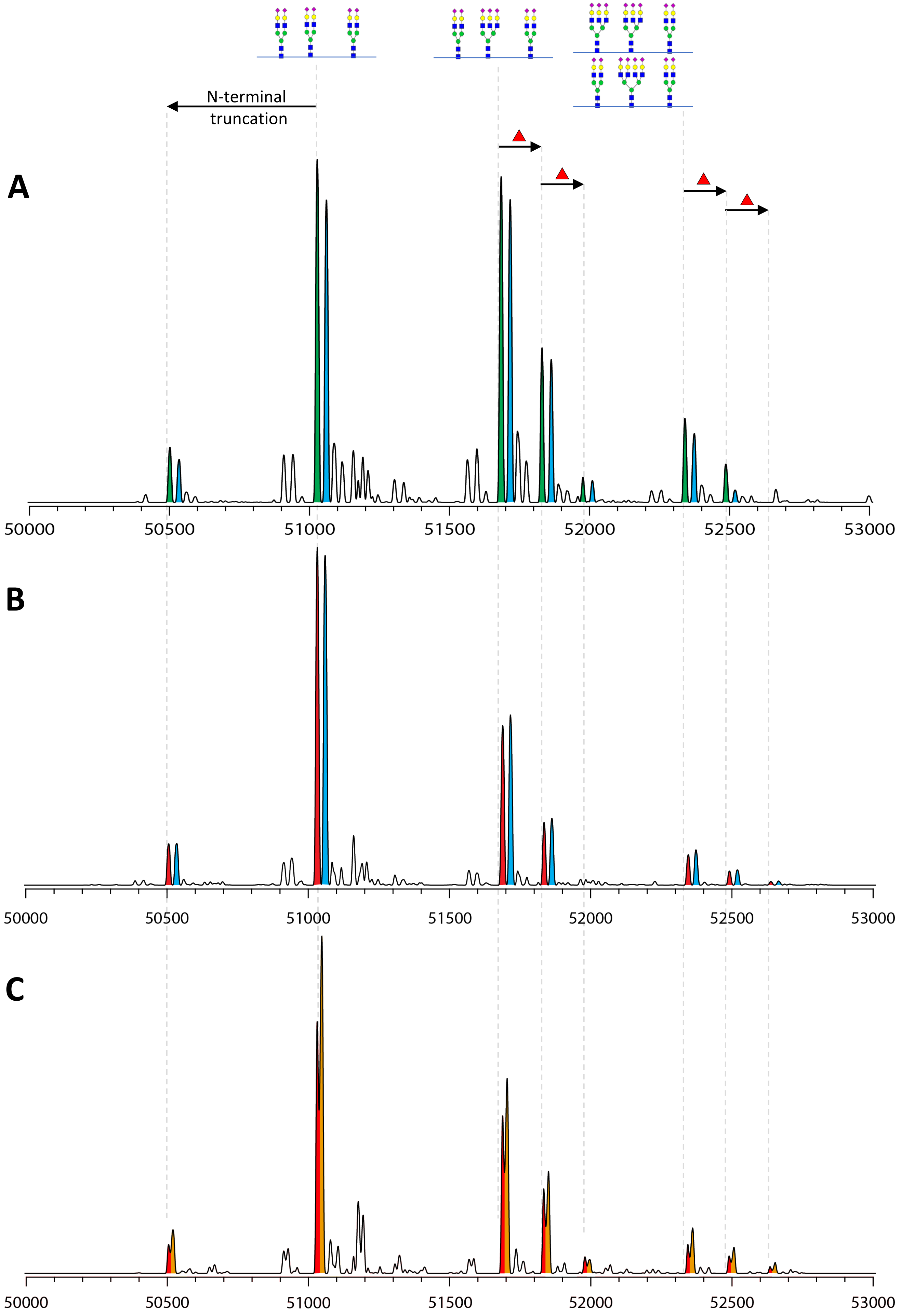
N-terminal
truncation
A
B
C
50000
50500
51000
51500
52000
52500
53000

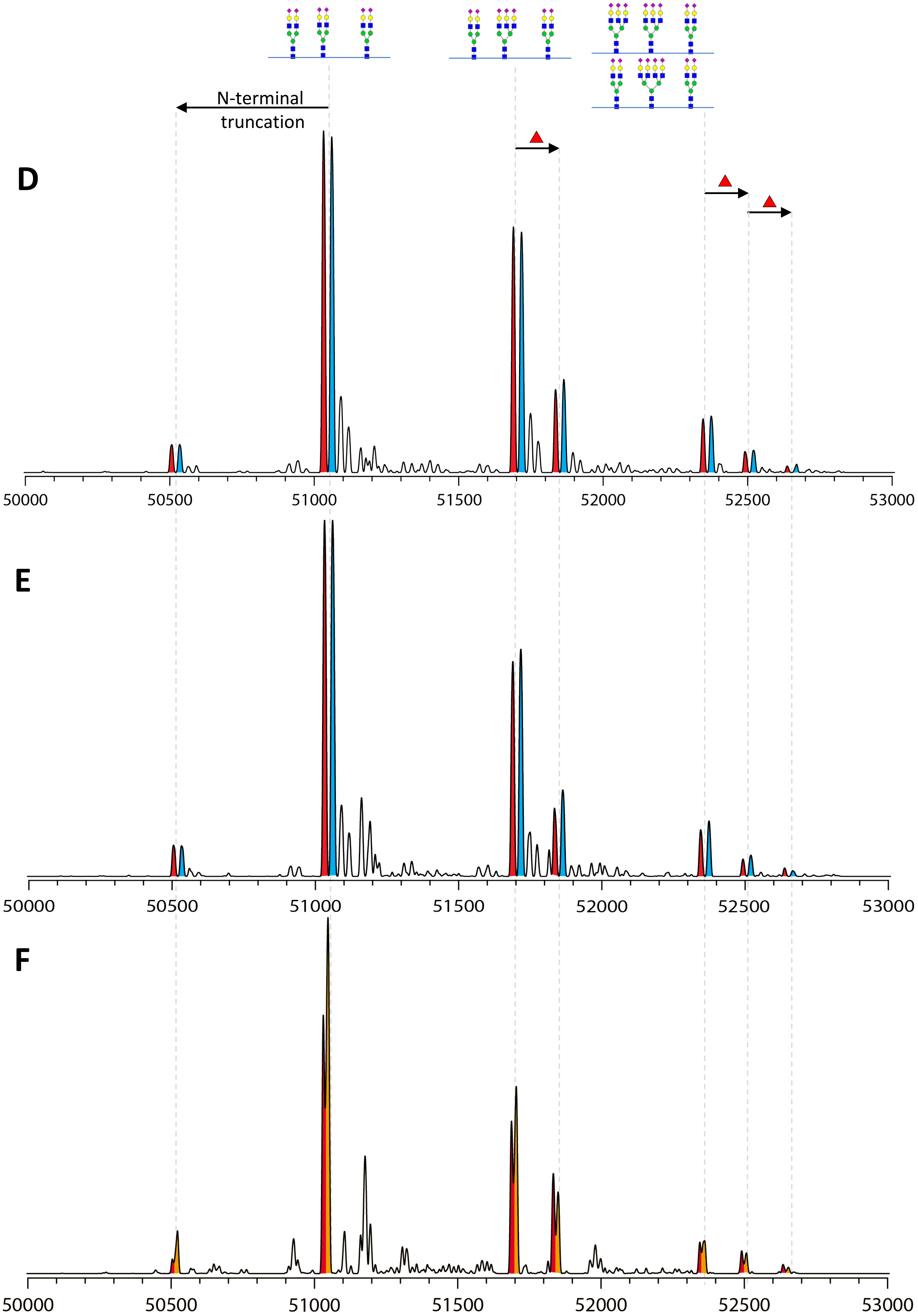
N-terminal
truncation
D
E
F
50000
50500
51000
51500
52000
52500
53000

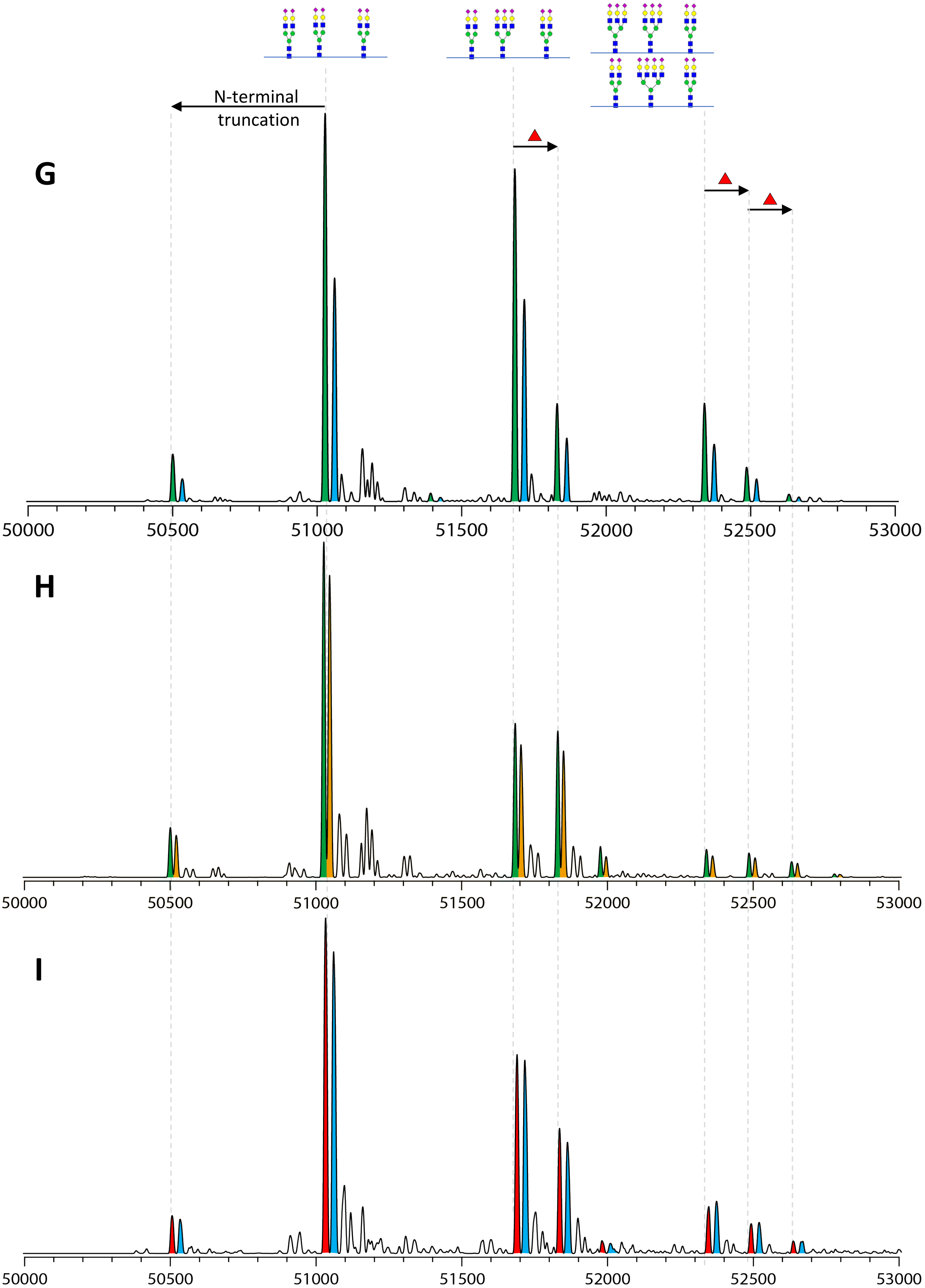
N-terminal
truncation
G
H
I
50000
50500
51000
51500
52000
52500
53000

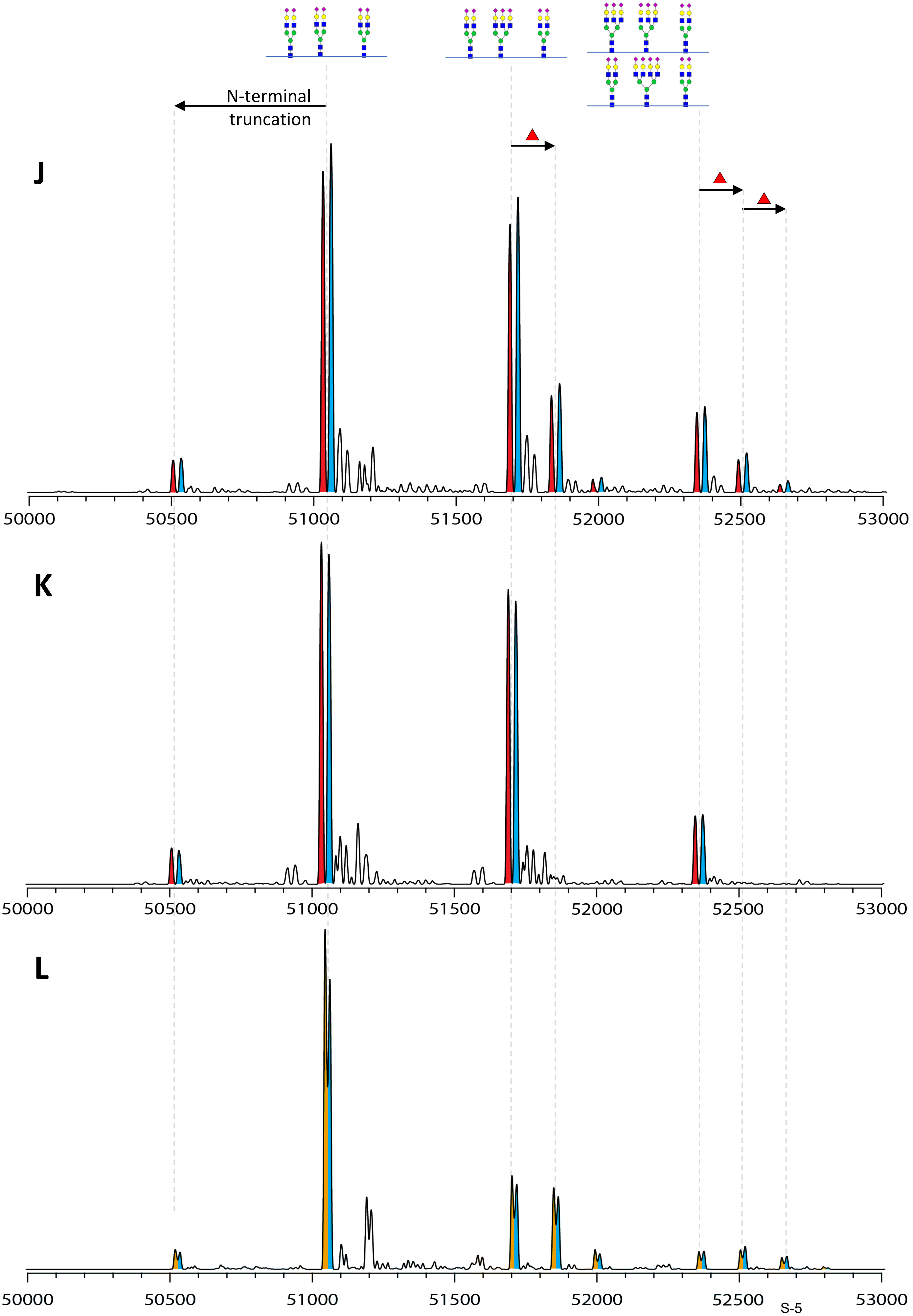
N-terminal
truncation
J
K
L
50000
50500
51000
51500
52000
52500
53000

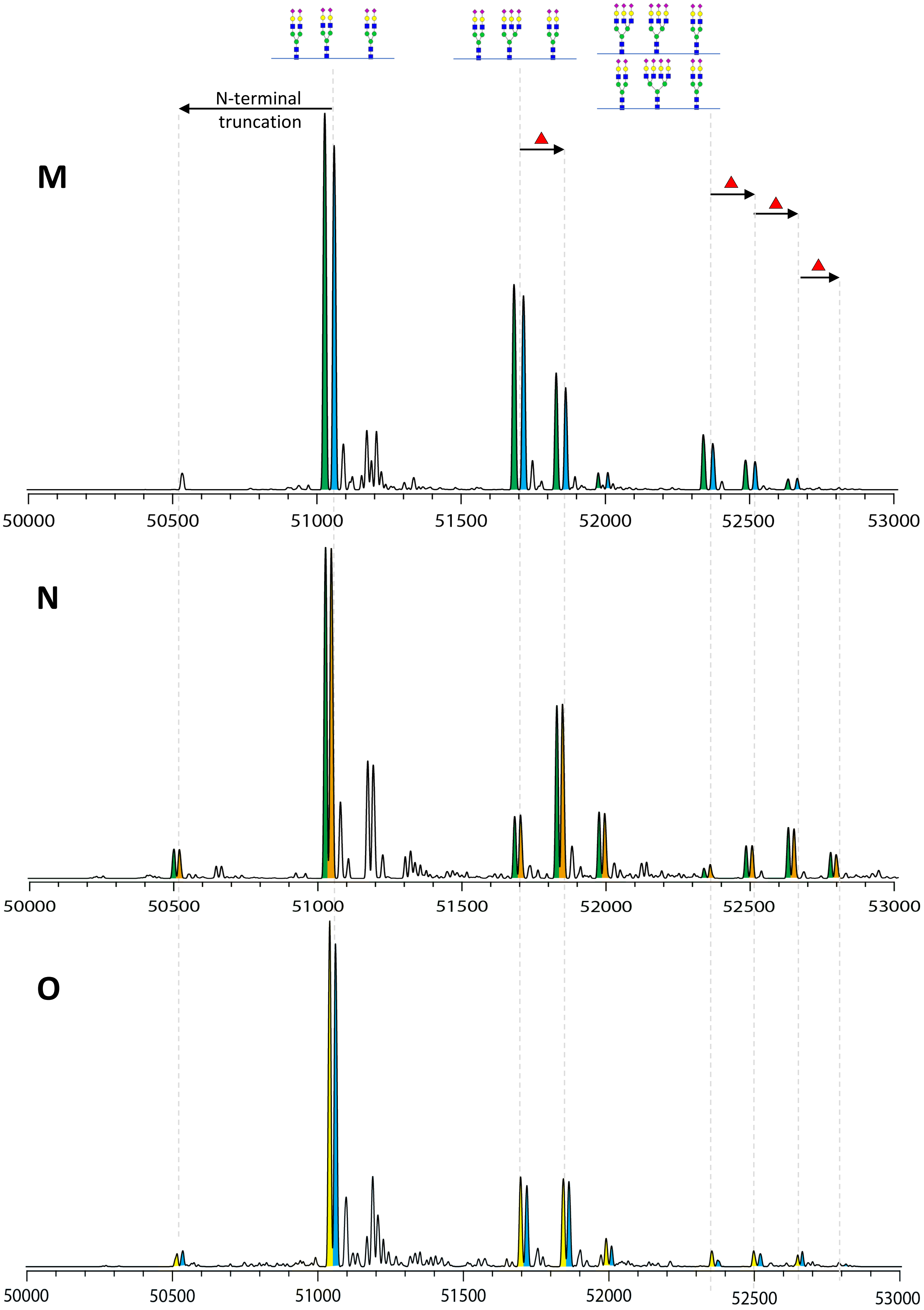
N-terminal
truncation
M
N
O
50000
50500
51000
51500
52000
52500
53000

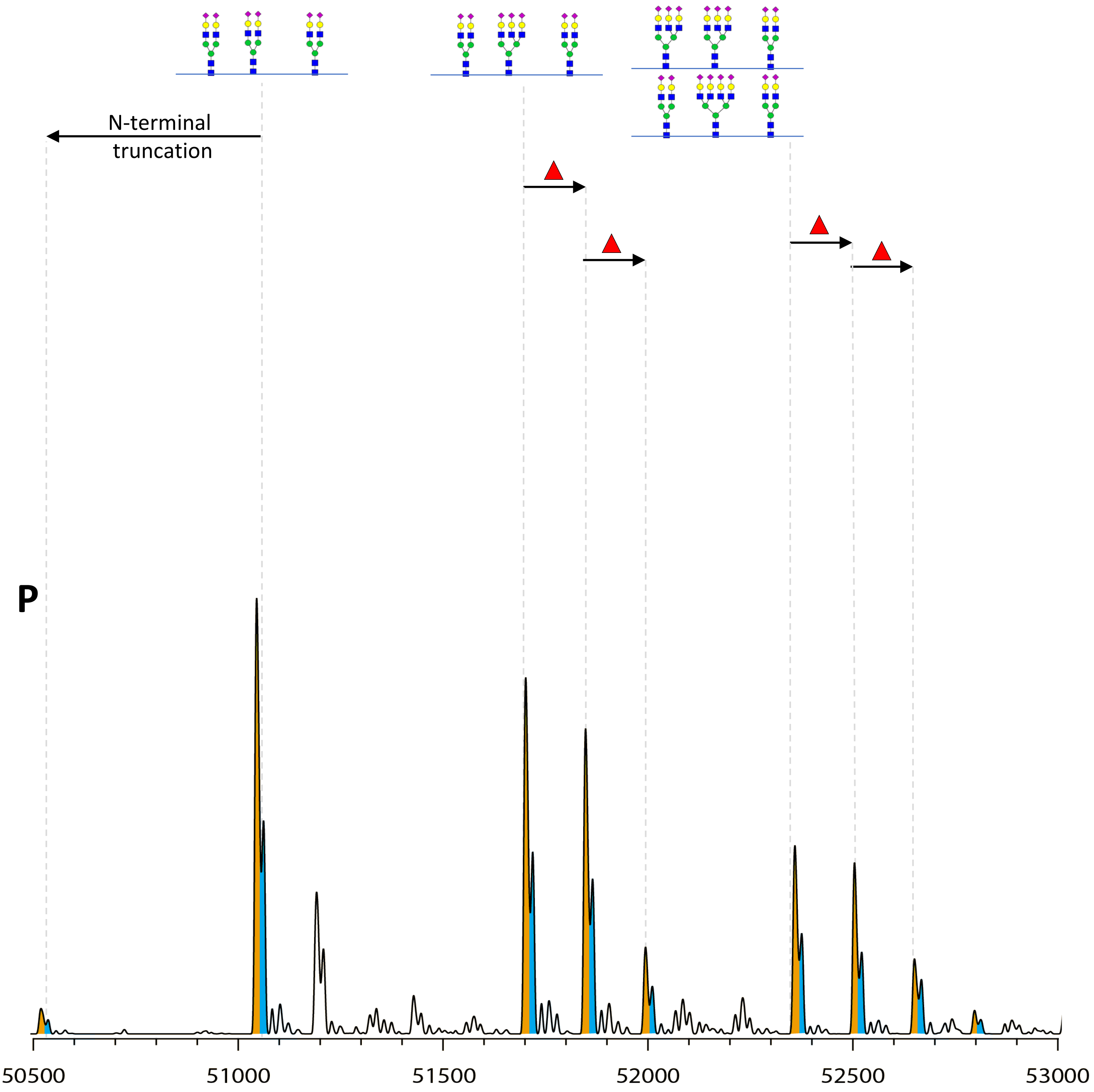
N-terminal
truncation
P
50500
51000
51500
52000
52500
53000

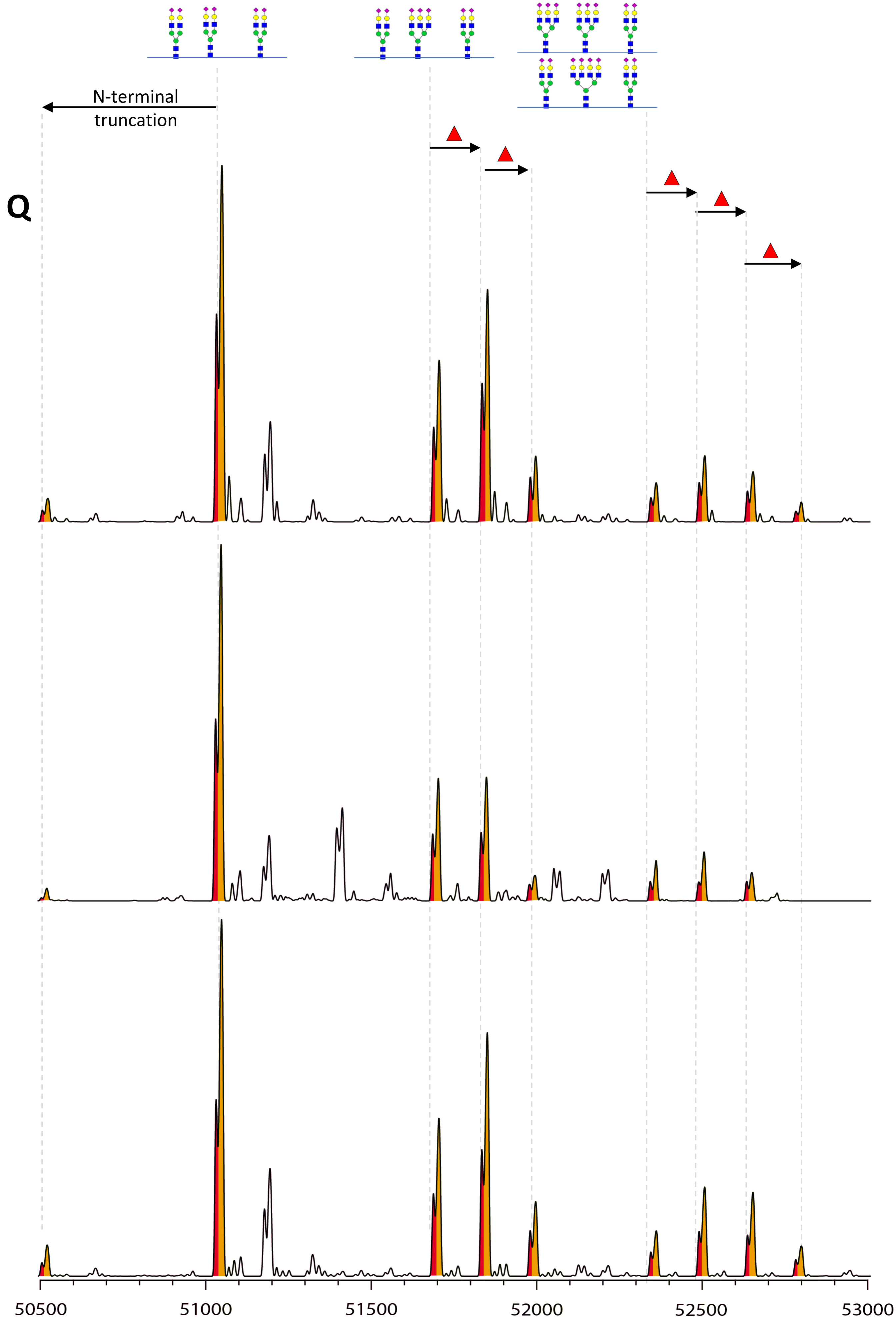
Q
N-terminal truncation
50500
51000
51500
52000
52500
53000

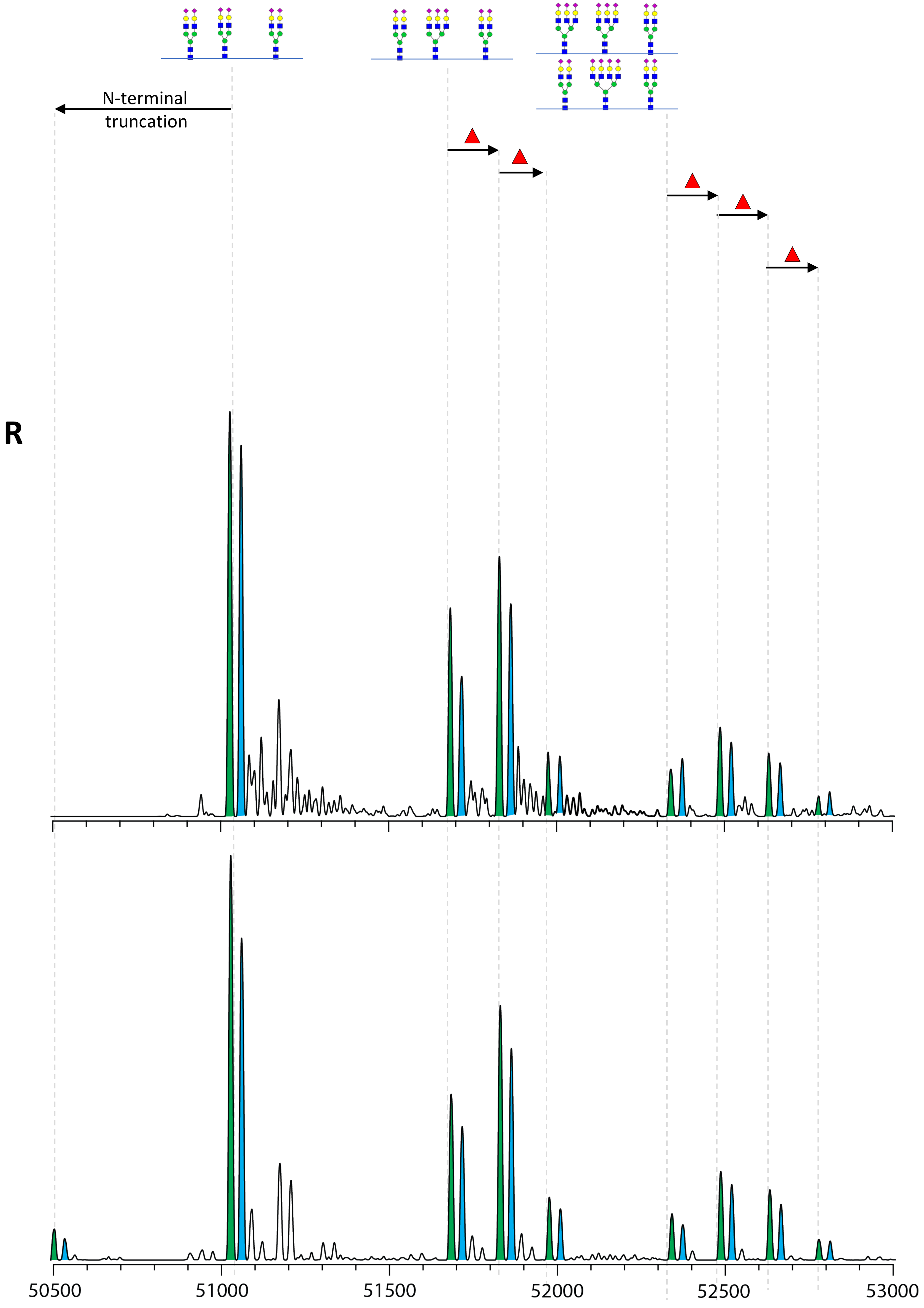

N-terminal
truncation
R
50500
51000
51500
52000
52500
53000

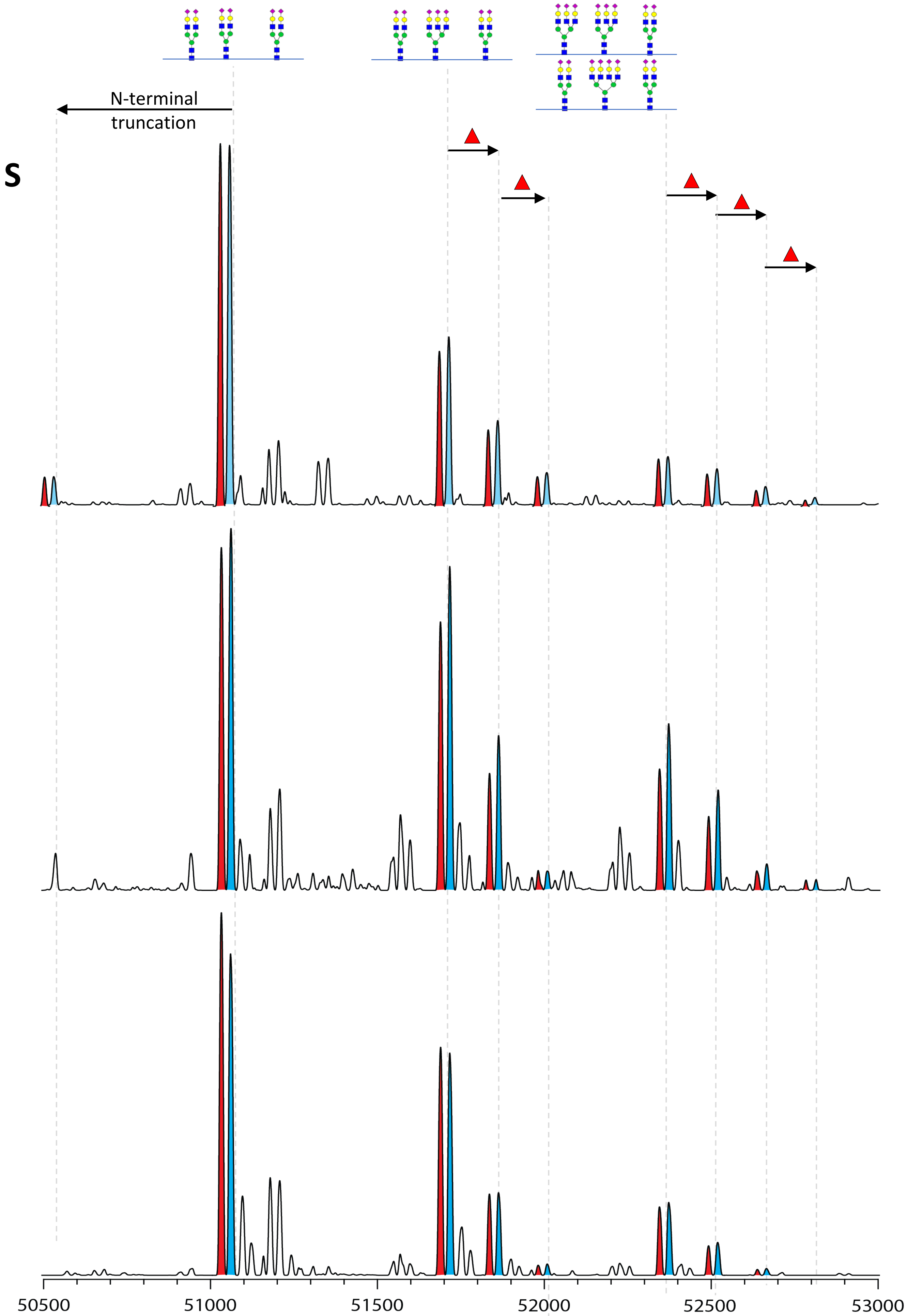

N-terminal
truncation
S
50500
51000
51500
52000
52500
53000

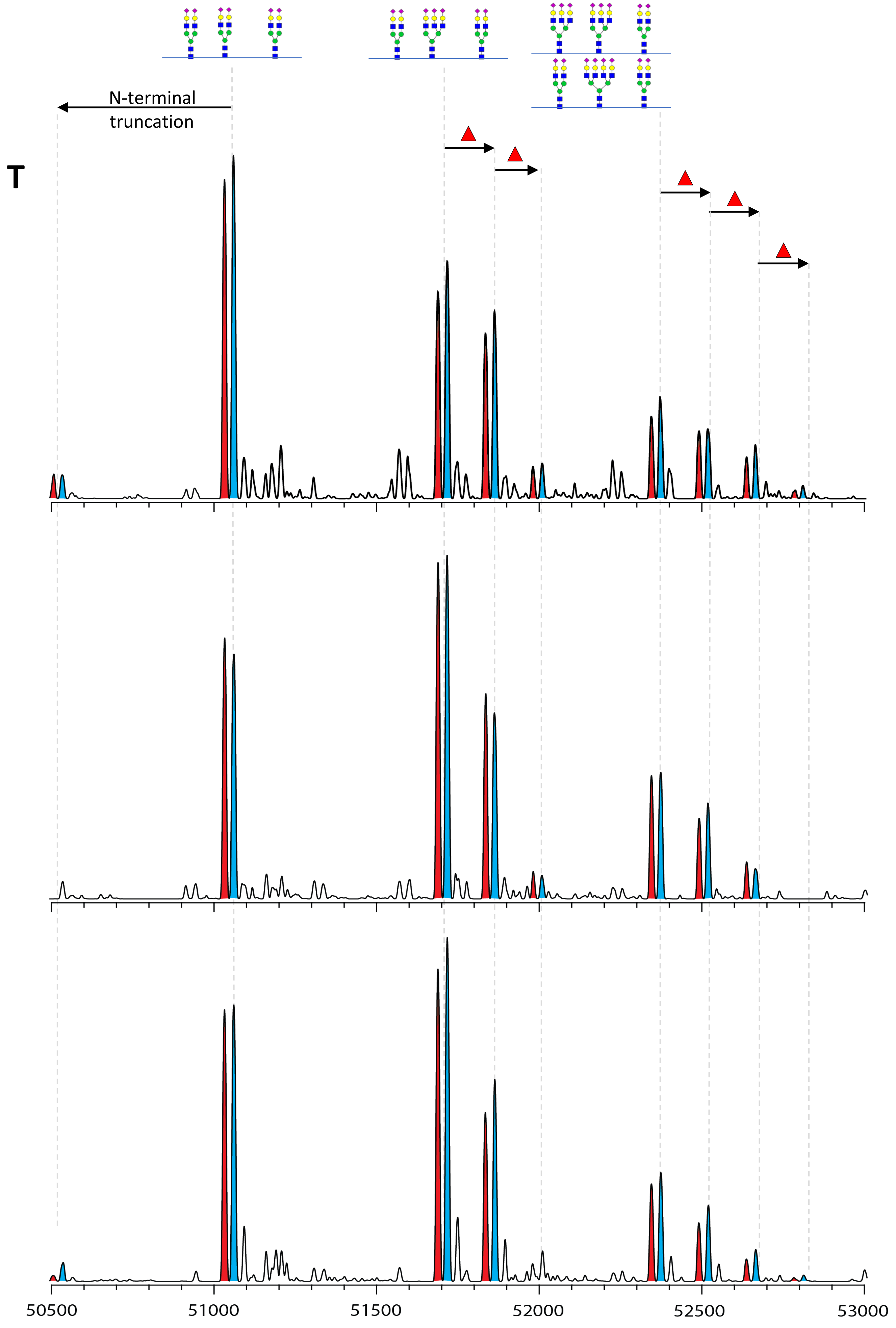
N-terminal
truncation
T
50500
51000
51500
52000
52500
53000

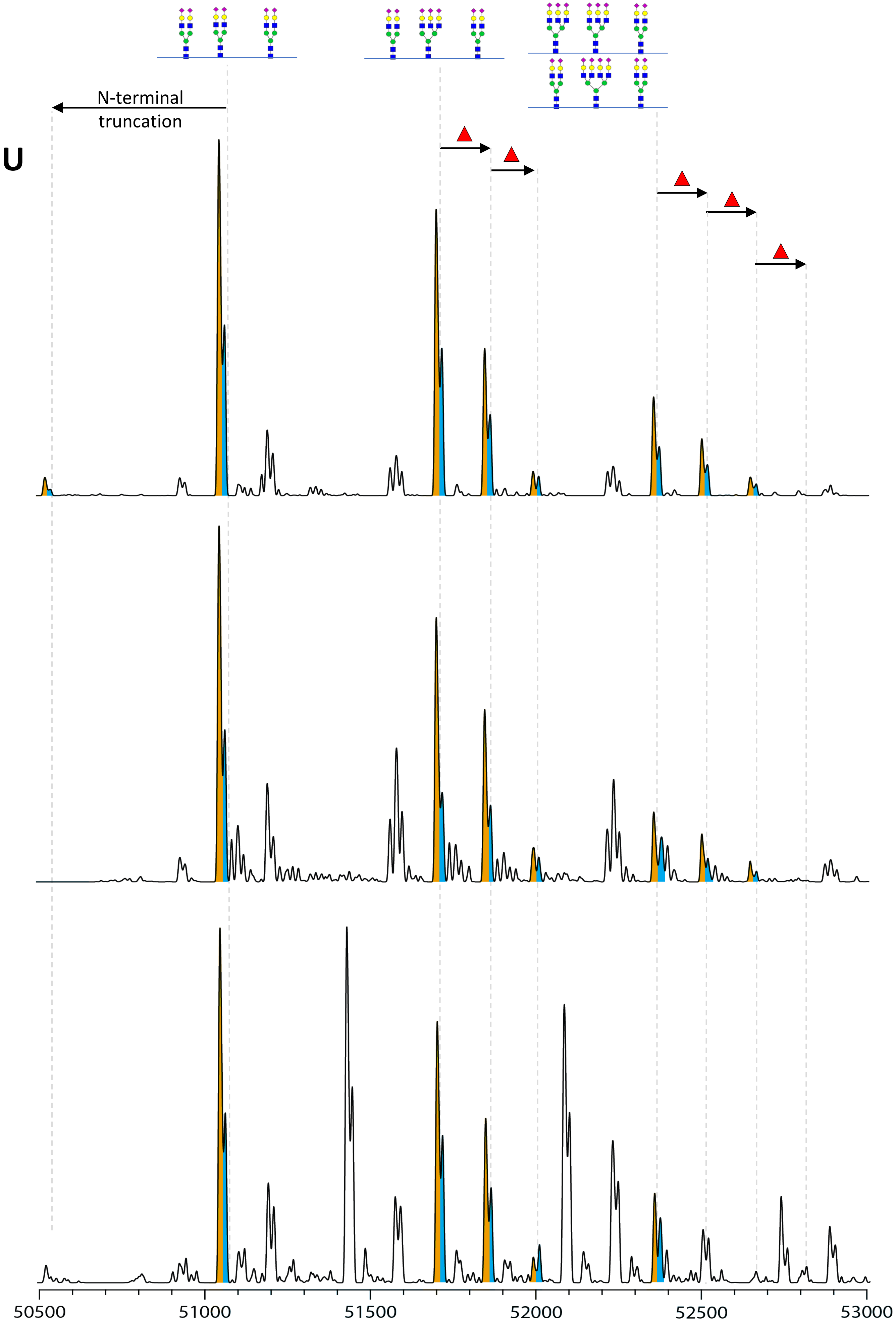
N-terminal
truncation
U
50500
51000
51500
52000
52500
53000

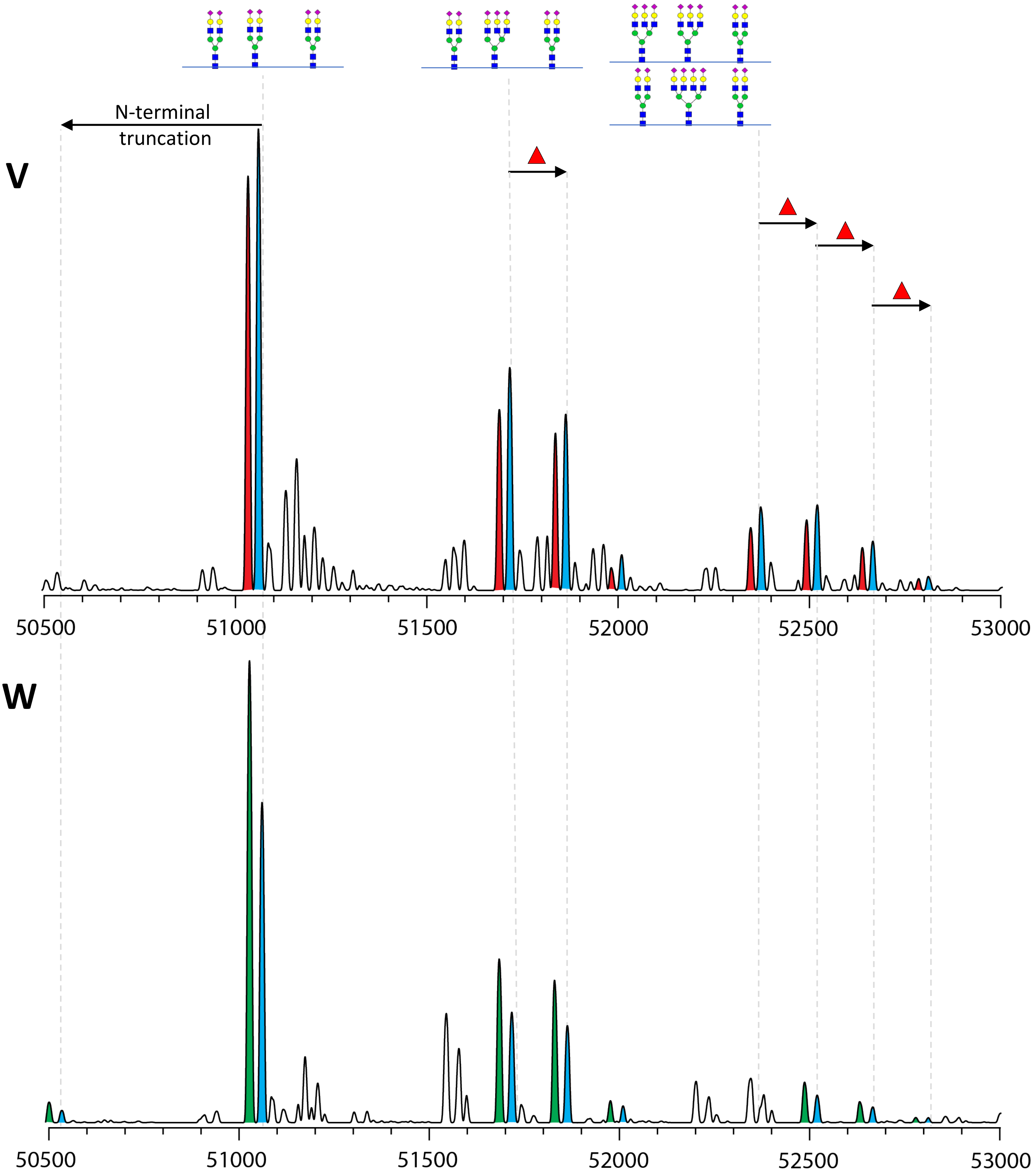
N-terminal
truncation
V
W
50500
51000
51500
52000
52500
53000

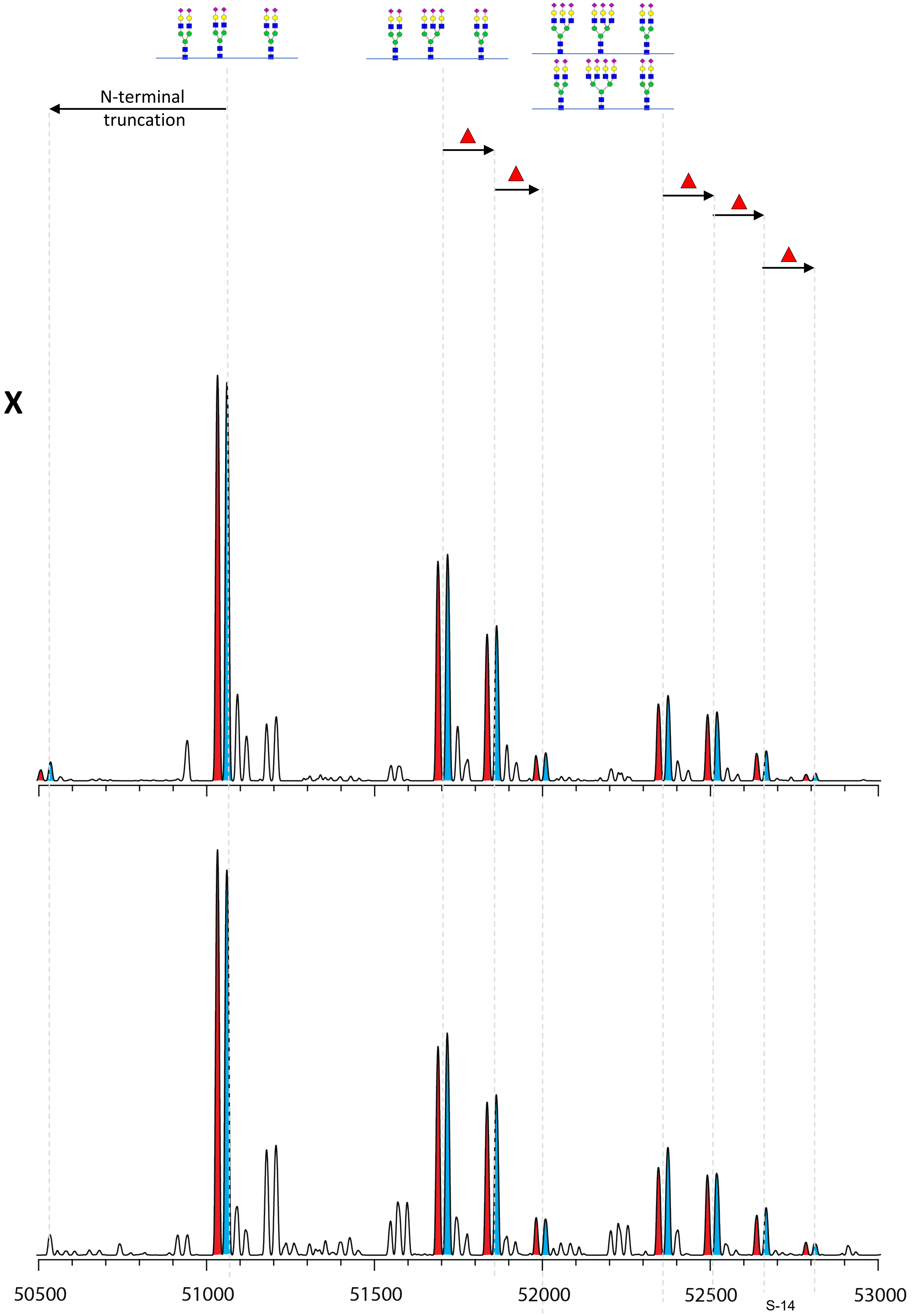
N-terminal
truncation
X
50500
51000
51500
52000
52500
53000

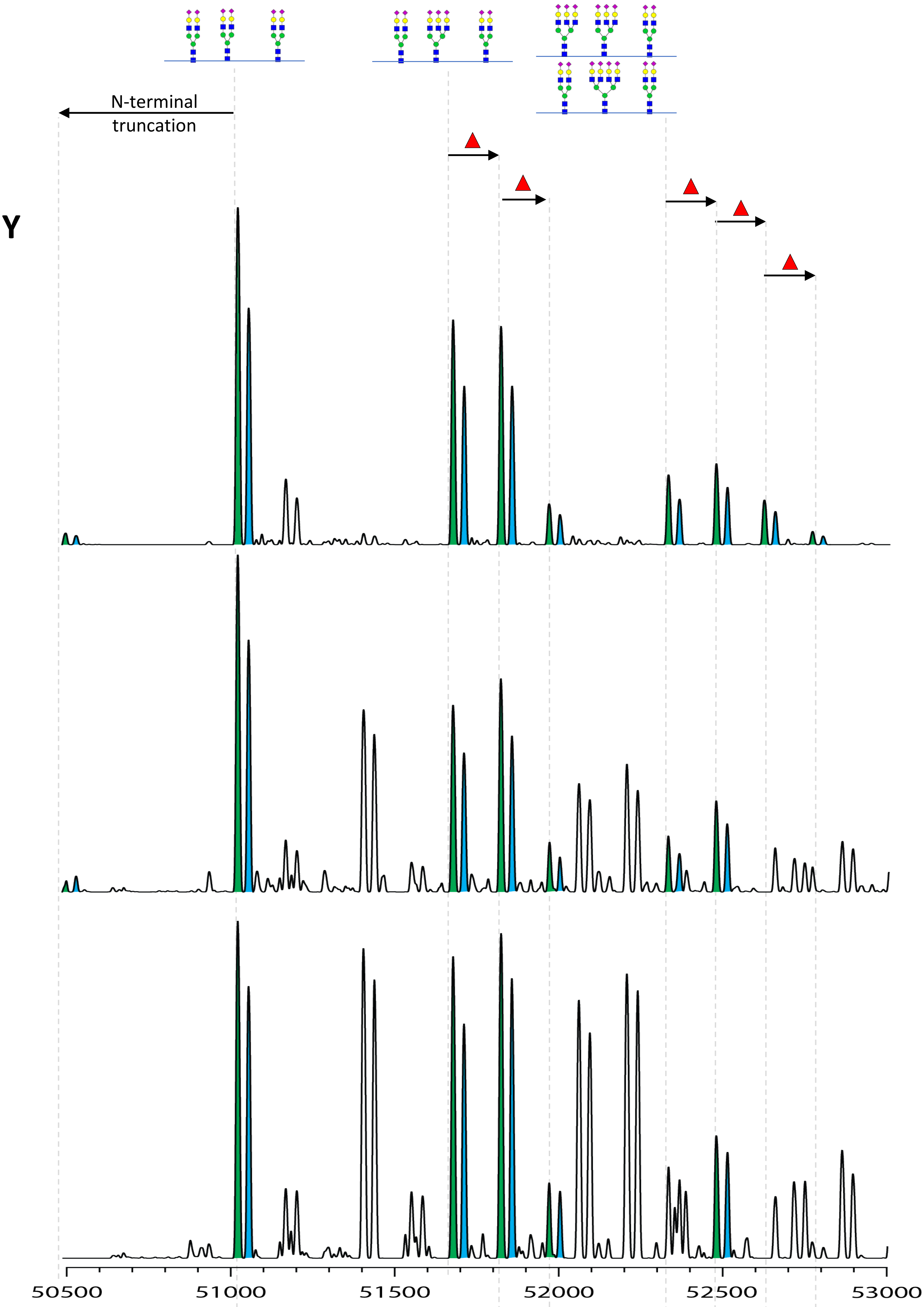
N-terminal
truncation
Y
50500
51000
51500
52000
52500
53000

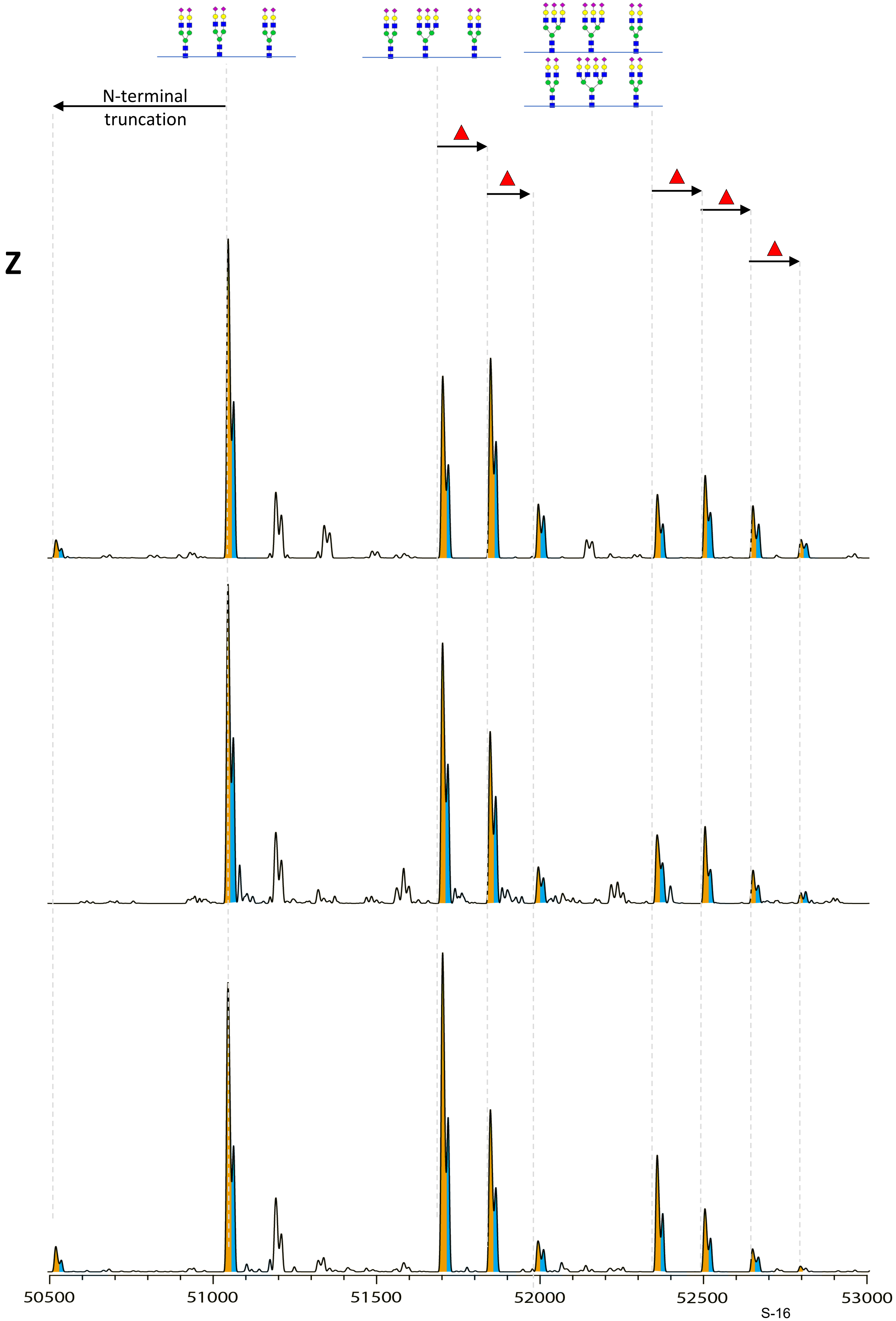

N-terminal
truncation
Z
50500
51000
51500
52000
52500
53000

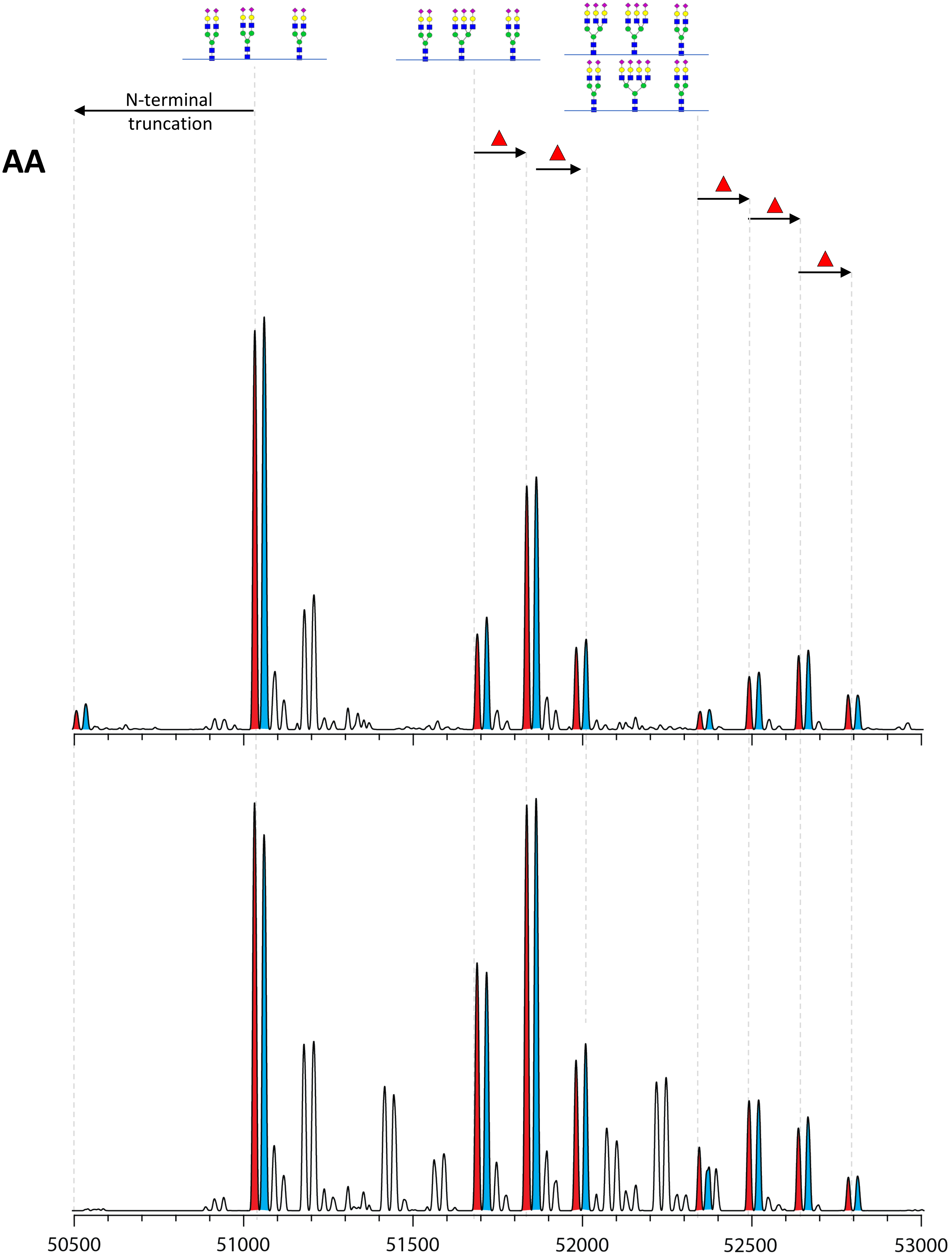
N-terminal
truncation
AA
50500
51000
51500
52000
52500
53000

Supplemental Figure S1: Annotated proteoform profiles of donors heterozygous for alpha-1-antitrypsin. Depicted is the zero-charge deconvoluted mass spectrum of A1AT. Peaks selected for quantification are colored in their respected genotype: blue = M1V, red = M1A, green = M2, orange = M3, and yellow = M4. These peaks are annotated by their most likely glycan composition and indicated with arrows are the N-terminal truncation and additional fucoses (red triangle), which can be located on the glycan core of on the antennae. **A)** donor 2; **B)** donor 5; **C)** donor 7; **D)** donor 8; **E)** donor 9; **F)** donor 14; **G)** donor 18; **H)** donor 23; **I)** donor 24; **J)** donor 25; **K)** donor 26; **L)** donor 27; **M)** donor 29; **N)** donor 30; **O)** donor 31; **P)** donor 32; **Q)** donor 33, samples a, b, and c from top to bottom; **R)** donor 34, samples a and b from top to bottom; **S)** donor 38, samples a, b, and c from top to bottom; **T)** donor 43, samples a, b, and c from top to bottom; **U)** donor 44, samples a, b, and c from top to bottom; **V)** donor 45; **W)** donor 46; **X)** donor 47, samples a, and b from top to bottom; **Y)** donor 48, samples a, b, and c from top to bottom; **Z)** donor 50, samples a, b, and c from top to bottom; **AA)** donor 51, samples a, and b from top to bottom.

Supplemental Table 1. Haplotype distribution and medical condition of each donor

sample ID	M1V	M1A	M2	M3	M4	condition
1		100				healthy
2	46.58		53.42			healthy
3	100					healthy
4		100				healthy
5	50.54	49.46				healthy
6			100			healthy
7		43.32		56.68		healthy
8	50.18	49.82				healthy
9	51.28	48.72				healthy
10			100			healthy
11	100					healthy
12			100			healthy
13	100					healthy
14		44.77		55.23		healthy
15		100				healthy
16	100					healthy
17	100					healthy
18	37.18		62.82			healthy
19		100				healthy
20		100				healthy
21		100				Hepatocellular carcinoma
22			100			Hepatocellular carcinoma
23			53.61	46.39		pancreatic carcinoma
24	48.27	51.73				pancreatic carcinoma
25	52.42	47.58				pancreatic carcinoma
26	49.16	50.84				Sepsis
27	46.96			53.04		Sepsis
28	100.00					Sepsis
29	47.79		52.21			Sepsis
30			49.98	50.02		Sepsis
31	47.97				52.03	Sepsis
32	34.11			65.89		COVID-19
33a		37.23		62.77		COVID-19
33b		34.30		65.70		COVID-19
33c		33.93		66.07		COVID-19
34a	46.16		53.84			COVID-19
34b	44.72		55.28			COVID-19
35a	100					COVID-19
35b	100					COVID-19
35c	100					COVID-19
36a	100					COVID-19
36b	100					COVID-19
36c	100					COVID-19
37a			100			COVID-19
37b			100			COVID-19
38a	50.79	49.21				COVID-19

38b	53.94	46.06			COVID-19
38c	48.83	51.17			COVID-19
39a	100				COVID-19
39b	100				COVID-19
40a			100		COVID-19
40b			100		COVID-19
40c			100		COVID-19
41a	100				COVID-19
41b	100				COVID-19
41c	100				COVID-19
42a	100				COVID-19
42b	100				COVID-19
43a	52.82	47.18			COVID-19
43b	49.30	50.70			COVID-19
43c	53.30	46.70			COVID-19
44a	33.90			66.10	COVID-19
44b	29.64			70.36	COVID-19
44c	36.94			63.06	COVID-19
45	53.91	46.09			COVID-19
46	40.76		59.24		COVID-19
47a	50.83	49.17			COVID-19
47b	50.66	49.34			COVID-19
48a	41.50		58.50		COVID-19
48b	41.86		58.14		COVID-19
48c	45.23		54.77		COVID-19
49a			100		COVID-19
49b			100		COVID-19
49c			100		COVID-19
50a	35.61			64.39	COVID-19
50b	35.73			64.27	COVID-19
50c	33.02			66.98	COVID-19
51a	51.57	48.43			COVID-19
51b	49.61	50.39			COVID-19
52a	100				COVID-19
52b	100				COVID-19
52c	100				COVID-19
